# Intercellular transmission of Seneca Valley virus mediated by exosomes

**DOI:** 10.1186/s13567-020-00812-x

**Published:** 2020-07-16

**Authors:** Guowei Xu, Shouxing Xu, Xijuan Shi, Chaochao Shen, Junhong Hao, Minhao Yan, Dajun Zhang, Zixiang Zhu, Keshan Zhang, Haixue Zheng, Xiangtao Liu

**Affiliations:** State Key Laboratory of Veterinary Etiological Biology, National Foot-and-Mouth Disease Reference Laboratory, Lanzhou Veterinary Research Institute, Chinese Academy of Agriculture Science, Lanzhou, 73004 China

**Keywords:** Exosomes, Infection, Seneca valley virus, Immune escape, Intercellular transmission

## Abstract

Seneca Valley virus (SVV) is a non-encapsulated single-stranded positive-strand RNA virus whose transmission routes have not yet been fully elucidated. Exosomes have been implicated in the intercellular transport of a variety of materials, such as proteins, RNA, and liposomes. However, whether exosomes can mediate SVV intercellular transmission remains unknown. In this study, we extracted exosomes from SVV-infected IBRS-2 cells to investigate intercellular transmission. Our results suggest that the intercellular transmission of SVV is mediated by exosomes. The results of co-localization and RT-qPCR studies showed that exosomes harbor SVV and enable the virus to proliferate in both susceptible and non-susceptible cells. Furthermore, the replication of SVV was inhibited when IBRS-2 cells were treated with interfering RNA Rab27a and exosome inhibitor GW4869. Finally, neutralization experiments were performed to further verify whether the virus was encapsulated by the exosomes that mediated transmission between cells. It was found that exosome-mediated intercellular transmission was not blocked by SVV-specific neutralizing antibodies. This study reveals a new transmission route of SVV and provides clear evidence regarding the pathogenesis of SVV, information which can also be useful for identifying therapeutic interventions.

## Introduction

The Seneca Valley virus (SVV) is a single-stranded positive-strand RNA virus that belongs to the *Senecavirus* genus and Picornaviridae family. SVV has a typical icosahedral symmetry and a genome 7.2 kb in length [1]. SVV was first discovered in 2002 in the PER.C6 cell line in Maryland, USA [2]. SVV mainly infects pigs, newborn piglets, fattening pigs, and other pigs of all ages; neutralizing antibodies have been found in other animals, such as cattle and sheep [3, 4]. Once infected, the clinical presentation is very similar to that of foot-and-mouth disease (FMD). The main symptoms are blisters and ulceration in the hoof and nose, as well as fever and anorexia [5]. In recent years, SVV has significantly affected the global pig industry due to the virus inducing blisters in pigs [6].

Exosomes are small vesicles with a diameter of 40–150 nm [7]. Most model cells secrete exosomes, which contain multiple substances, including large amounts of proteins and nucleic acids, and transport substances to various cells [8–10]. The virus enters cells through endocytic pathways during the process of exosome formation and completes its assembly and release [11]. Hepatitis A (a picornavirus) and Hepatitis C viral exosomes can spread their DNA and escape the immune response [12]. Therefore, we suspect that exosomes may be an essential mediator of SVV transmission between cells.

In the present study, we aimed to determine whether exosomes can mediate SVV transmission. First, we extracted exosomes from IBRS-2 cells with (SVV-exo) and without (mock-exo) SVV infection. After identification of the extracted exosomes, we introduced the extracted exosomes into 293T and IBRS-2 cells. The results suggested that SVV carried by exosomes can proliferate in these cells. We then inhibited the secretion and production of exosomes, which resulted in the inhibition of SVV proliferation. Finally, we found that SVV carried by exosomes was not blocked by SVV neutralizing antibodies. This study provides critical information regarding the pathogenesis of SVV and its antiviral mechanisms.

## Materials and methods

### Cell culture and viruses

To obtain a cell culture supernatant for exosome extraction, we used IBRS-2 cells as a model. IBRS-2 cells were cultured in Dulbecco’s modified Eagle’s medium (DMEM) supplemented with 10% fetal bovine serum (FBS), 100 IU/mL penicillin, and 100 mg/mL streptomycin. The cells were cultured in an incubator maintained at 37 °C with a CO_2_ concentration of 5%. In January 2017, SVV strain CH-FJ-2017 (GenBank Accession number: KY74510) was isolated from Fujian, China, at our lab; this same strain was used throughout the present study. SVV-expressing green fluorescent protein (SVV-GFP) was constructed at our laboratory.

### Exosome isolation and purification

To obtain exosomes secreted by SVV-infected cells, we inoculated SVV into IBRS-2 cells and collected the supernatants at specific times after infection. SVV was isolated previously, as described later in the text, and preserved at our lab (China Reference Laboratory Network for FMD) [13]. IBRS-2 cells were incubated in a 150-mm culture dish until they attained confluency (Corning, New York, USA). The culture supernatant was then discarded, the cells were washed with PBS, and FBS-free DMEM was added. SVV (0.05 TCID_50_) was inoculated, and PBS was used as a control. After 1 h of incubation, SVV was discarded and replaced with DMEM containing 2% exosomes-depleted FBS. The cell culture supernatant was collected after 36 h of culture. To further separate and purify the collected supernatant, we performed differential centrifugation with the collected supernatant. The following centrifugation processes were conducted at 4 °C. The collected supernatant was centrifuged at 500 × *g* for 5 min to remove larger fragments and cells, and the supernatant was then collected and centrifuged at 2000 × *g* for 10 min to further remove cell debris. The collected supernatant was centrifuged at 12 000 × *g* for 45 min to remove cells. The large vesicles were collected and filtered through a 0.22-µm filter. Finally, the collected supernatant was centrifuged at 120 000 × *g* for 2 h in an ultracentrifuge (Thermo Scientific Sorvall WX100), and the precipitates were resuspended in 500 μL of PBS. To further purify the extracted exosomes, we used a CD63 antibody-labeled exosomes isolation kit (Miltenyi Biotec, Bergisch Gladbach, Germany).

### Transmission electron microscopy (TEM)

Direct morphological observation of the characteristics of exosomes is crucial for exosome identification [14] . Therefore, we analyzed the extracted exosomes using TEM (Hitachi H-7000FA, Tokyo, Japan). After observation, we first extracted the exosomes using a TEM 200 copper mesh (EMS 80100-Cu US) and then stained the exosomes with phosphoric acid dock for 2 min. After drying under an incandescent lamp, electron microscopy was used to observe the extracted exosomes, and the observed voltage was 80 kV.

### Western blot analysis

Western blot (WB) analysis was performed using the following protocols. Briefly, purified exosomes were lysed with a radio-immunoprecipitation assay buffer (Santa Cruz Biotechnology, Dallas, TX, USA), and the cleared lysate was collected by centrifugation for protein separation on 12% sodium dodecyl sulfate–polyacrylamide gel electrophoresis. After electrophoresis, the separated proteins were transferred onto 0.45-μm polyvinylidene difluoride membranes (Millipore, USA). Next, the membranes were blocked for 1 h with 10% fat-free milk in Tris-buffered saline containing Tween-20 (TBST). The blots were then incubated with primary antibodies at 4 °C overnight. Primary antibodies for CD63 (Abcam, Cambridge, UK), CD9 (Abcam, Cambridge, UK), Alix (Cell Signaling Technology, Waltham, MA, USA), and SVV polyclonal antibody (prepared at our laboratory) were used. After washing three times with TBST, the membranes were incubated with horseradish peroxidase-labeled secondary antibodies (Proteintech, Chicago, IL, USA) for 2 h at room temperature. Finally, the proteins were visualized with a clarity-enhanced chemiluminescence WB substrate (Bio-Rad Laboratories, Hercules, CA, USA).

### Nanoparticle tracking analysis (NTA)

The mean size and size distribution profile of exosomes that were isolated and purified from SVV-infected or control-treated IBRS-2 cell culture supernatants were analyzed as previously described [15, 16]. In brief, the samples were diluted at a ratio of 1:1000 in PBS containing 0.05% Tween-20 in a total volume of 1.0 mL. Measurements were performed in triplicate using standard settings (refractive index = 1.331, viscosity = 0.89, and temperature = 25 °C). Data analysis was performed using NTA 3.2 software (Malvern Panalytical Ltd., Malvern, Worcestershire, UK), and samples were evaluated using the Nanosight NS300 (Malvern Panalytical Ltd., Malvern, Worcestershire, UK).

### Analysis and quantification of SVV RNA

For the PCR detection of SVV RNA, total RNA from exosomes (RNase added to purified exosomes followed by incubation for 1 h at 37 °C before RNA extraction) and cells were extracted using a total exosome RNA and protein isolation kit (Life Technologies, USA) according to the manufacturer’s instructions. Total RNA from cell culture samples were isolated with the E.Z.N.A. total RNA kit I (Omega Bio-Tek) to quantify RNA copies of SVV in SVV-infected or exosome-treated cells. Detection of the number of copies of extracted RNA was performed using the Real-Time One-Step RT-PCR reagent (Takara). The reaction system was as follows: 2X One-Step RT-PCR Buffer III 10 μL, TaKaRa Ex Taq HS (5 U/μL) 0.4 μL, Prime Script RT Enzyme Mix II 0.4 μL, PCR forward primer (10 μM) 0.4 μL, PCR reverse primer (10 μM) 0.4 μL, SVV-3D probe 0.8 μL, total RNA 2 μL, and RNase-free dH_2_O 5.2 μL (PCR primers and the SVV-3D probe were provided by our laboratory). The reaction times and temperatures of the PCR were 42 °C for 15 min (1 cycle) and 40 cycles of 94 °C for 10 s, 57 °C for 30 s, and 72 °C for 30 s. The Applied Biosystems 7300 Real-Time PCR System (Thermo Fisher) was used.

### Si-Rab27a transfection and quantification of Rab27a and Alix mRNA

Interfering RNA Rab27a (100 pmol) was transfected into IBRS-2 cells using liposome 2000; at the same time, the same dose of si-scr (same bases, different base sequences) was used as a control. SVV was inoculated 24 h after transfection, and cells were harvested 24 h after SVV inoculation. Total RNA from cell samples were isolated using the E.Z.N.A. total RNA kit I (Omega Bio-Tek). Reverse transcription of RNA into cDNA was performed using Prime Script TM RT Master Mix (Takara). The reaction system was as follows: 5X PrimeScript RT Master Mix (Perfect Real Time) 2 μL, total RNA 5 μL (200 ng), and RNase-free dH_2_O 3 μL. The reaction temperature and time of the PCR were 37 °C for 15 min and 85 °C for 5 s (reverse transcription reaction), respectively. Using *GAPDH* as an internal reference gene, RT-qPCR was performed using TB Green TM Premix Ex TaqTM II (Takara). The reaction system was as follows: TB Green Premix Ex Taq II (Tli RNaseH Plus) 10 μL, PCR forward primer (10 μM) 0.8 μL, PCR reverse primer (10 μM) 0.8 μL, DNA (< 100 ng) 2 μL, and sterilized water 6.4 μL.

### Exosome treatment with SVV-specific neutralizing antibody

IBRS-2 cells were plated into 12-well cell culture plates, and the cells were replaced with serum-free DMEM once the cells reached 70%–80% confluency. SVV-exo and SVV were simultaneously diluted to obtain concentrations ranging from 10^−1^ to 10^−4^. The diluted SVV-exo and SVV were incubated with the SVV neutralizing antibody for 1.5 h at 37 °C and then added to the prepared IBRS-2 cells. At the same time, SVV-exo and SVV that were not incubated with the SVV neutralizing antibody were used as controls. The cells were cultured in a 5% CO_2_ cell culture incubator at 37 °C for 24 h. The cells and culture supernatants were used to detect the copy number of SVV.

## Results

### Isolation and characterization of exosomes extracted from SVV-infected IBRS-2 cells

**Figure 1:**
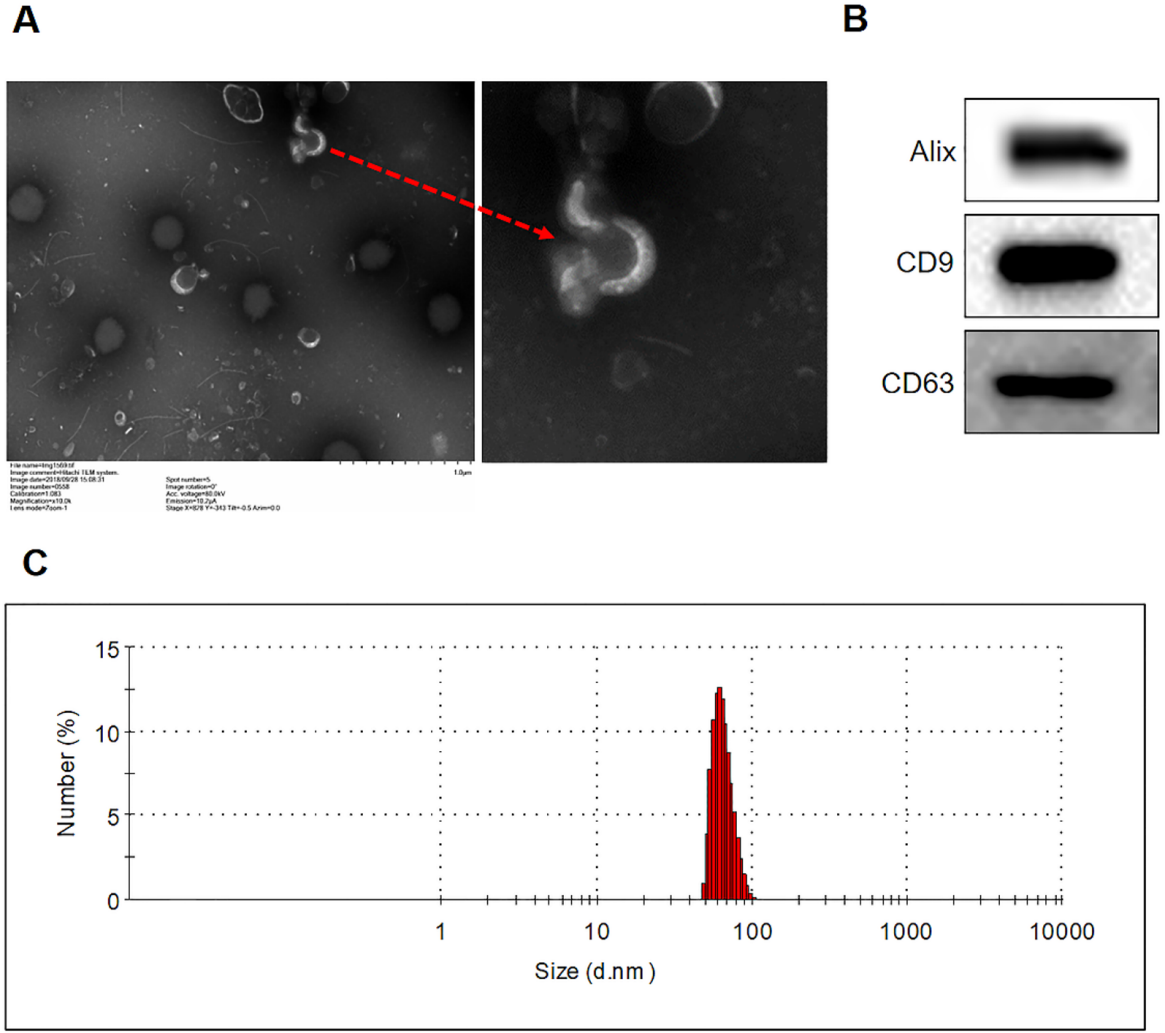
**Isolation and characterization of exosomes extracted from SVV-infected IBRS-2 cells. A** Transmission electron microscopic observation of SVV-exo after negative staining with phosphotungstic acid. **B** Exosomes were extracted from SVV-infected IBRS-2 cells, and identified by western blot with antibodies directed against Alix, CD9, and CD63. **C** The Zeta Sizer Nano ZS was used to measure the particle size distribution. The samples were diluted 1: 1000 in PBS containing 0.05% Tween-20 to a total volume of 1.0 mL. The detection was repeated three times with the standard settings (refractive index = 1.331, viscosity = 0.89, and temperature = 25 °C). Each sample was analyzed five times, and the counts were averaged.

IBRS-2 cells were infected with SVV, and then exosomes were extracted from those infected cells (SVV-exo), as well as the non-infected cells (Mock-exo). Further purification was performed with the use of CD63-antibodies. Cup-shaped lipid bilayer vesicles of representative exosome were observed by TEM (Figure 1A). WB analysis was performed to identify exosomes (protein marker Alix, CD63, and CD9 were used). The results showed that SVV-exo contained the exosome-associated proteins Alix, CD9, and CD63 (Figure 1B). Exosome size was also evaluated, and it was found that the exosomes were mainly distributed in the range of 50–150 nm (Figure 1C). These results indicate that the morphology and particle size of the exosomes extracted in this study is consistent with that previously reported [17].

### The spread of SVV mediated by exosomes in susceptible and non-susceptible cells

**Figure 2:**
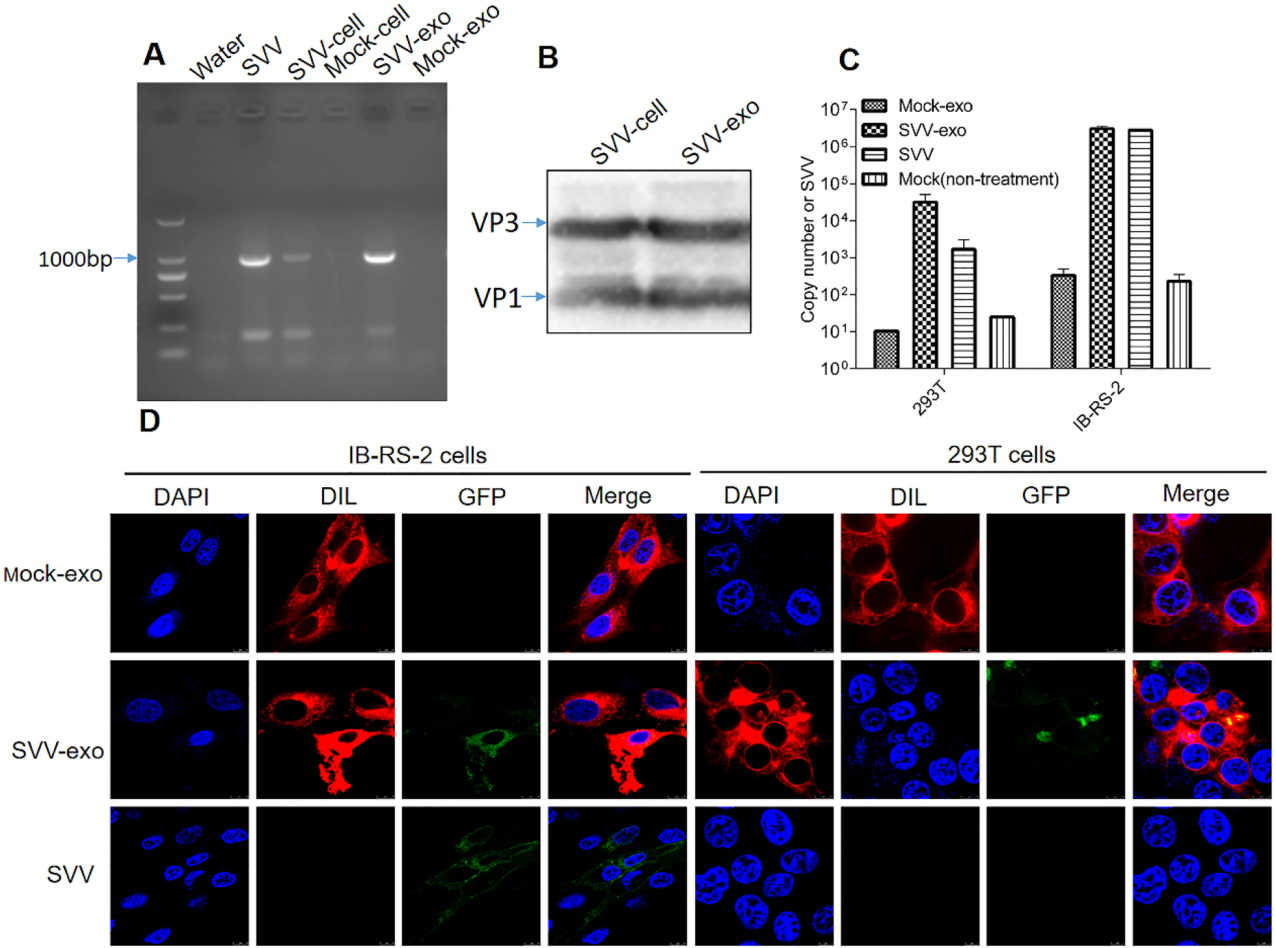
**Exosomes mediate the spread of SVV in susceptible and non-susceptible cells. A** SVV VP1 gene was identified in SVV-exo by the RT-qPCR, at the same time, SVV, SVV-infected IBRS-2 cells (Cell-svv) was used positive control, normal IBRS-2 cells (Cell-mock), and mock-exo were used as negative controls. **B** SVV polyclonal antibody used to detect SVV protein levels in SVV-exo, also use cell-svv as a control. **C** SVV-exo were incubated with 293T and IBRS-2 cells, and at the same time, mock-exo, SVV with the same viral load as SVV-exo, and PBS were used as controls. The virus copy number was detected by RT-qPCR 24 h after incubation. **D** Exosomes extracted from SVV-GFP-infected cells (SVV-GFP-exo) were stained with DIL, washed twice by ultracentrifugation (Avoid light during this experiment), and incubated in 293T and IBRS-2 cells; same procedures were followed for SVV-GFP as a control. Eight hours after incubation, the nuclei were stained with DAPI, and fluorescence was observed.

The SVV gene sequence in exosomes was detected by RT-PCR to verify whether the extracted exosomes contained SVV. SVV gene sequences were found in SVV-infected IBRS-2 cells and SVV-exo (Figure 2A). Moreover, SVV proteins were detected in exosomes by WB (Figure 2B). It is known that exosomes can mediate the spread of various pathogens [18]. To explore the role of exosomes in SVV transmission, we explored whether SVV carried by exosomes can proliferate on susceptible (IBRS-2) and non-susceptible (293T) cells. IBRS-2 cells and 293T cells were incubated with SVV-exo, and SVV was used as a positive control, mock-exo and normal cells-mock (non-treatment) were used as negative controls. The copy number of SVV was detected by RT-qPCR. According to the experimental results, the copy number of SVV in the SVV-exo group was increased in both 293T and IBRS-2 cells when compared with the negative control group, and the copy number in IBRS-2 cells was higher than that in 293T cells (Figure 2C).

Whether exosomes could transport SVV into susceptible and non-susceptible cells was investigated by fluorescently labeling exosomes and SVV. Exosomes were extracted and purified from the culture supernatant of SVV-GFP infected IBRS-2 cells (SVV-GFP-exo). The DIL-stained exosomes were introduced into IBRS-2 and 293T cells, mock-exo and SVV-GFP were introduced as controls. Fluorescence signals were observed under a laser confocal microscope. The results showed that red fluorescence (DIL) was observed in both IBRS-2 and 293T cell membranes, and the GFP carried by SVV-GFP-exo co-localized with DIL. In addition, only red fluorescence was observed in the mock-exo group (Figure 2D). Henceforth, these results indicate that the spread of SVV could be mediated by exosomes in susceptible and non-susceptible cells.

### Inhibition of exosomes secretion suppresses the proliferation of SVV

**Figure 3:**
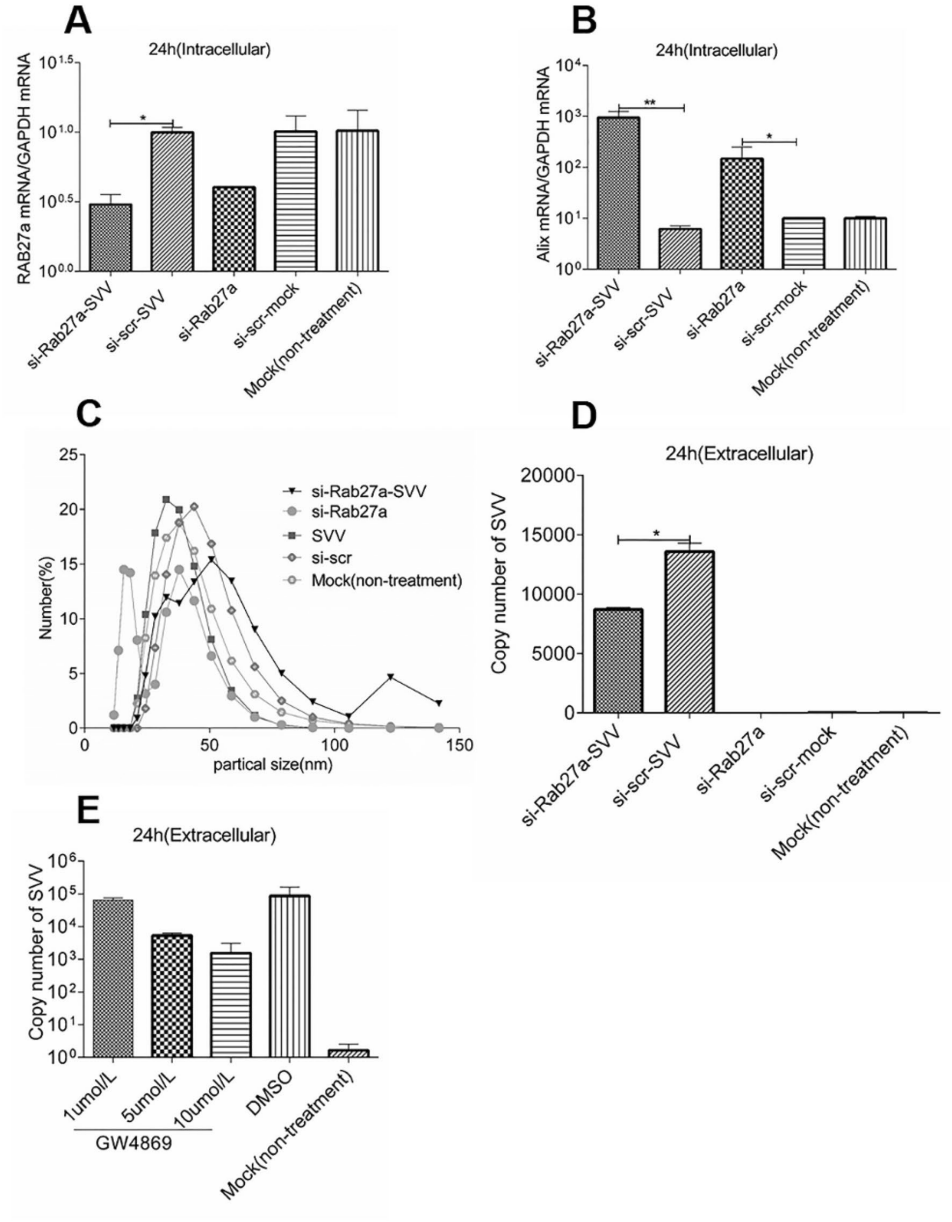
**Interfering with Rab27a inhibits secretion of exosomes and thus suppresses SVV transmission.** Si-Rab27a interfering RNA (100 pmol) was transfected into IBRS-2 cells using liposome 2000. SVV was inoculated 24 h after transfection (si-Rab27a-SVV), the controls were transfected with Rab27a but not inoculated with SVV (si-Rab27a), and transfected with si-scr inoculated with SVV (si-scr-SVV), and transfected of si-scr but not inoculated with SVV(si-scr), and untreated cells-mock (non-treatment). **A** The expression of Rab27a mRNA in IBRS-2 cells was detected by RT-qPCR, and used GAPDH as a reference gene. **B** The expression of Alix mRNA in the cell was detected by RT-qPCR, and used GAPDH as a reference gene. **C** Exosomes were extracted from the culture supernatants of SVV-infected IBRS-2 cells, and the number of exosomes was detected by the NTA method. **D** The copy number of extracellular SVV in IBRS-2 cells was detected by RT-qPCR. Significance was calculated using a two-tailed *t* test and labeled as *P < 0.05 and **P < 0.01 in graphs. **E** IBRS-2 cells were infected with SVV for 1.5 h and then incubated with 1, 5, and 10 µmol of GW4869 for 36 h. An equal volume of DMSO was used as a control, and cells without any treatment were used as a negative control. The extracellular SVV copy number was detected using RT-qPCR.

Previous studies have found that Rab27a [19] and Rab35 [20] regulate exosome secretion. Thus, to verify whether intracellular Rab27a expression was downregulated after si-Rab27a transfection, si-Rab27a was transfected into IBRS-2 cells, and Rab27a mRNA levels were evaluated by RT-qPCR. The results showed that the mRNA expression of intracellular Rab27a was significantly downregulated in si-Rab27a-transfected cells compared with that in the control group (Figure 3A). Previous work has confirmed that the ESCRT (Endosomal Sorting Complexes Required for Transport) pathway can affect exosome secretion. Syntenin interacts directly with Alix via the LYPX (n) L motif to regulate the intraluminal budding of ILV and Alix is often used as a biological protein marker for exosomes [21]. In order to investigate whether the number of exosomes in the transfected cells did change, the mRNA expression level of the exosome protein marker Alix was evaluated. The results showed that the expression of Alix was significantly upregulated in IBRS-2 cells treated with si-Rab27a compared with that of control (IBRS-2 cells treated with si-scr that same base as si-Rab27a but different sequence) (Figure 3B). The number of exosomes in the cell culture supernatant of si-Rab27a transfected IBRS-2 cells was evaluated using the NTA method. The results showed that the number of exosomes in the cell culture supernatant decreased significantly after si-Rab27a transfection (Figure 3C).

An investigation into the observed changes in the number of exosomes and whether they affected the copy number of SVV in cells was also conducted. RT-qPCR was performed to detect the copy number of SVV in the cell culture supernatant of the si-Rab27a and control group. The results showed that extracellular SVV copy number was significantly decreased compared with those in the control group (Figure 3D). GW4869 was reported to reduce exosome release carrying PLP (proteolipid protein) by inhibiting neutral sphingomyelinase (nSMase) [22]. IBRS-2 cells were infected with SIBRS-2 cells were infected with SVV; the infected cells were treated with exosomes secretion inhibitor GW4869; simultaneously, an equal volume of DMSO was used as a control. The copy number of extracellular SVV was detected by RT-qPCR. The results show that the copy number of extracellular SVV is significantly decreased after GW4869 treatment compared with the control group and is dose-dependent (Figure 3E).

### Exosomes promote the proliferation of SVV in susceptible cells

**Figure 4:**
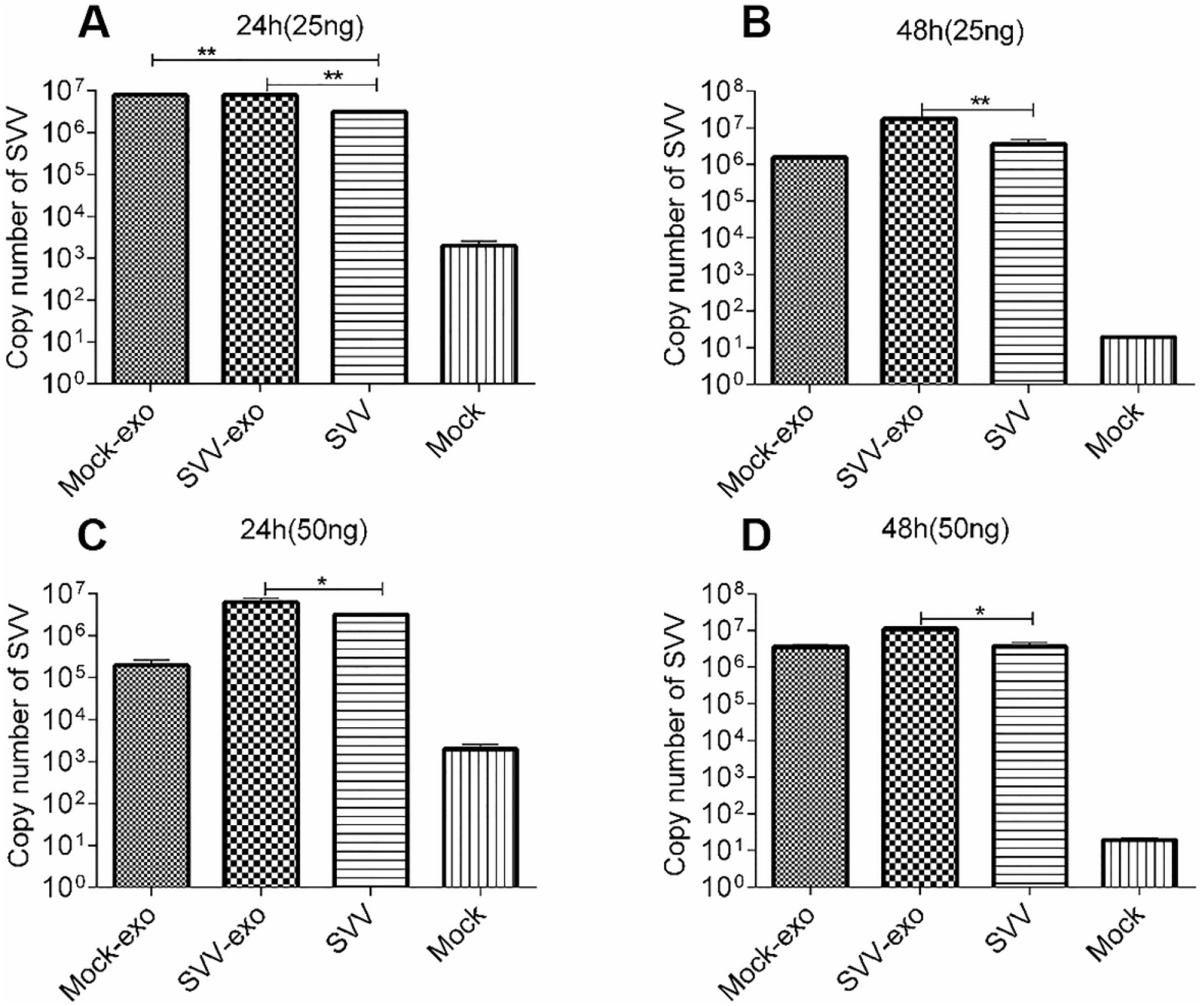
**Exosomes promote the proliferation of SVV in IBRS-2.** Exosomes were extracted from SVV-infected IBRS-2 cells and normal IBRS-2 cells separately, and the A280/A260 method was used to determine the protein concentration of SVV-exo and mock-exo. SVV-exo and mock-exo were treated with RNase for 1 h at 37 °C before they were incubated into IBRS-2 cells. Different doses of SVV-exo and mock-exo was incubated into IBRS-2 cells, SVV with the same SVV copy number as SVV-exo infected IBRS-2 cells and mock-exo, normal IBRS-2 cells as control, 8 h after incubation, the same copy number of SVV were infected again in the SVV-exo experimental group, mock-exo and SVV control groups, respectively. SVV copy number was detected using RT-qPCR at 24 h and 48 h after SVV infection. **A** The exosome protein concentration was inoculated at a dose of 25 ng, and the viral load was measured 24 h after SVV infection. **B** The exosome protein concentration was inoculated at a dose of 25 ng, and the viral load was measured 48 h after SVV infection. **C** The exosome protein concentration was inoculated at a dose of 50 ng, and the viral load was measured 24 h after SVV infection. **D** The exosome protein concentration was inoculated at a dose of 25 ng, and the viral load was measured 48 h after SVV infection. This experiment was repeated three times, all data represent mean ± SD, n = 3 for each group. Significant difference was calculated using two-tailed t-test and labeled as *P < 0.05 and **P < 0.01 in graphs.

In the continued attempt to elucidate whether SVV-exo promotes SVV proliferation, the protein concentration of SVV-exo and mock-exo was determined, and the exosomes (RNase was added to purify exosomes) were restored to IBRS-2 cells at two different doses, at the same time. A SVV that had the same SVV copy number as SVV-exo and mock-exo was used as a control. The SVV was re-inoculated after incubation, and untreated cells were also used as a control-mock (non-treatment). SVV RNA copy number was detected by RT-qPCR at 24 h and 48 h, respectively, after SVV inoculation. The results showed that the SVV copy number was significantly higher in mock-exo and SVV-exo groups than that of the SVV control group at 24 h after SVV infection (Figure 4A). Moreover, the SVV-exo group had more SVVs at 48 h after SVV infection compared to the other two groups (Figure 4B). Nevertheless, when the exosomes dose was 50 ng, the SVV copies in the SVV-exo group was significantly higher than that in the SVV control group, at both 24 h and 48 h after SVV infection (Figure 4C-D). The above results indicate that SVV-exo promotes SVV proliferation in IBRS-2 cells.

### SVV infection mediated by exosomes is not inhibited by SVV NAbs

**Figure 5:**
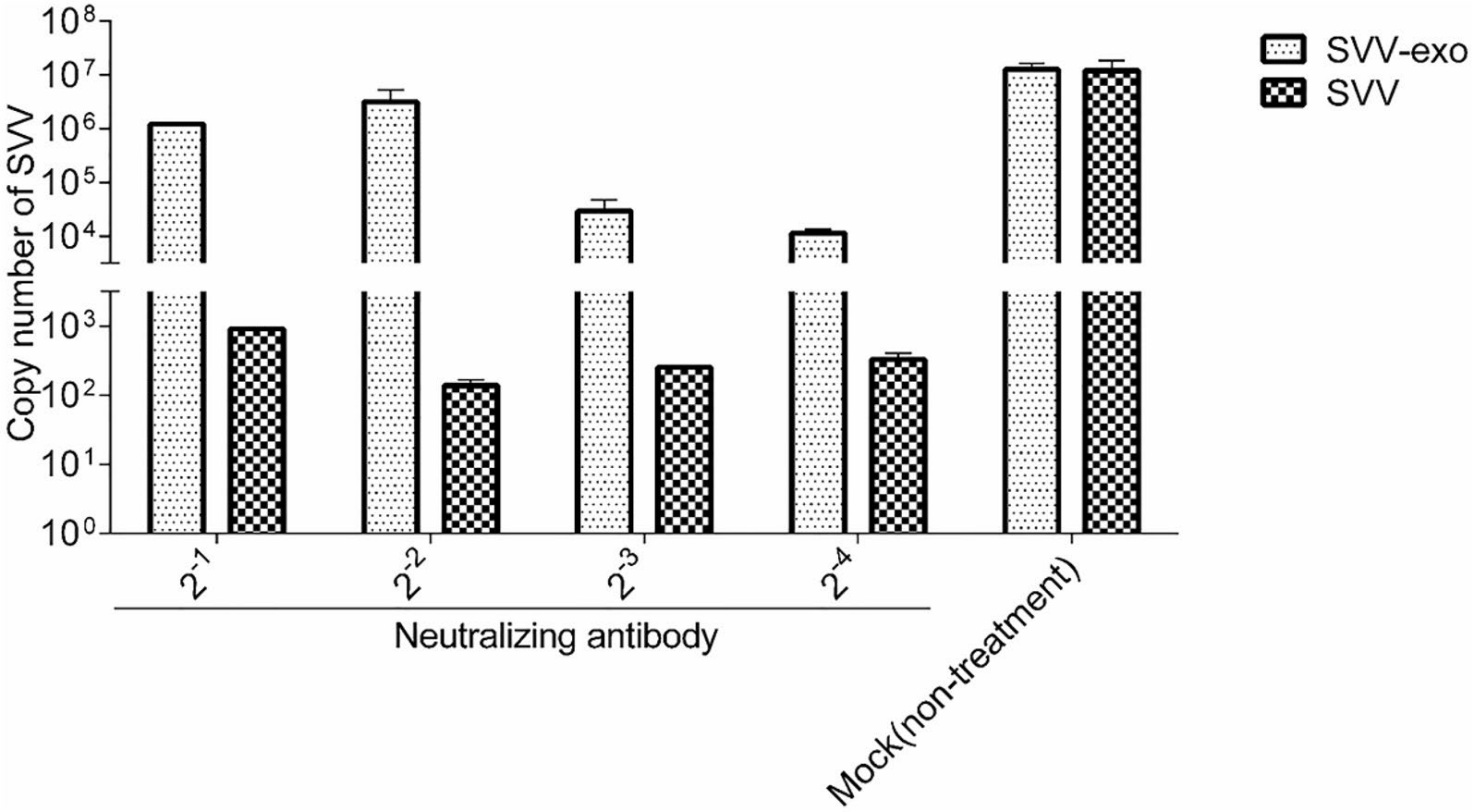
**Exosome-mediated SVV infection is not blocked by SVV-specific neutralizing antibodies (NAbs).** Purified SVV-positive exosomes and SVV were diluted and then the diluted exosomes and SVV were incubated with SVV-specific NAbs separately (the titer of serum neutralization against SVV was > 1:1024, determined by VNT) for 1 h. Exosomes and SVV without any treatment were used as a control. Then, IBRS-2 cells were exposed to the NAbs-treated exosomes or SVV for 2 h. The exosomes or viruses were washed off with PBS at 37 °C, and the medium was replaced with fresh maintenance medium for 24 h. RT-qPCR was used to evaluate SVV replication in IBRS-2 cells.

A neutralization experiment was performed to exclude the influence of the possible appearance of SVV in the exosome suspension. SVV-exo and SVV were diluted ten times and then subsequently incubated with a SVV neutralizing antibody for 1.5 h. Exosomes and SVV were inoculated into IBRS-2 cells, and the copy number of SVV in the inoculated cells was detected by RT-qPCR (Figure 5). The results show that the proliferation of SVV in SVV-exo on IBRS-2 cells is not affected by SVV neutralizing antibodies. It suggests that SVV particles are encapsulated in the exosomes, and the SVV infection is mediated by SVV wrapped in exosomes.

## Discussion

Exosomes are thought to mediate the transmission of various intracellular molecules which can influence cell function, such as proteins, RNA, and miRNA. Although, the contents of the exosomes that are secreted by infected recipient cells changed, they could still contain viral RNA, proteins, and virions which mediate virus transmission. In the current study, we found that the transmission of SVV in cultured cells could be mediated by exosomes. Although the SVV-exo was treated with RNase before inoculating cells, it was impossible to prove that all naked virions outside the exosomes were cleared. Therefore, SVV and SVV-exo were inoculated with the same SVV copy number into SVV non-susceptible cells (293T cells). The results showed that the SVV-exo group had a higher copy number on non-susceptible cells than the SVV control group, indicating that SVV-exo mediated the proliferation of SVV. Henceforth, the results suggest that exosomes can serve as delivery vectors for pathogen-associated molecules [23]. In addition, their role in viral infections is attracting more and more attention [24, 25]. Some viruses may be able to evade the immune response as they spread through the body by exosomes [26]. During the process of exosome formation, some viruses, mainly in the endocytic pathway, enter the cell through exosomes resulting in direct cell entry without the need for cell membrane receptors [12, 26, 33]. From the above results, it can be postulated that exosomes mediate the transmission of SVV. However, whether exosomes play an important role in the transmission of SVV in host cells remains to be fully elucidated.

Furthermore, an investigation was conducted to explore whether the inhibition of exosome secretion affects exosome proliferation. Previous studies have shown that silencing the expression of Rab27a and Rab27b reduces exosome secretion of CD63, CD81, and MHC class II, and the downregulation of Rab27a effector Slp4 and Slac2b also inhibits exosome secretion [27, 28]. Therefore Rab27a siRNA was synthesized in the lab and transfected into IBRS-2 cells. The results showed that both the number of exosomes secreted by IBRS cells and extracellular SVV had decreased significantly. GW4869 is a neutral sphingomyelinase inhibitor that is known to inhibit the biosynthesis of ceramides and promotes exosome secretion by triggering the germination of exosomes to MVB [29]. When the secretion of IBRS-2 cell exosomes was inhibited by interfering with Rab27a and using GW4869, the amount of SVV in the cell culture supernatant decreased significantly. Therefore, the proliferation of SVV is affected by the secretion of exosomes, which further illustrates that exosomes mediate the transmission of SVV. Previous studies have shown that the formation of cellular infectious prion is significantly impaired when the HRS-ESCRT-0 subunit is silenced and that the overall secretion of exosomes is also impaired [30]. HCV secretion from host cells utilizes the Hrs-dependent exosomal pathway in which the viral assembly is also involved [31]. The findings here are consistent with those reported in the literature.

There are some limitations to this work; whether there are naked virus particles in the extracted exosomes has not been shown. Further, an immunomagnetic bead method is used to purify and extract the exosomes, and the exosomes are treated with RNase. However, the exosomes, as they are a type of lipid vesicle, may rupture before entering the cell. Nonetheless, IBRS-2 cells were incubated with SVV-exo and SVV of the same copy number. They were then incubated with more SVV, and the number of SVV copies after SVV-exo incubation was significantly higher than that of the SVV control group. This further suggests that exosomes mediate SVV transmission and promote SVV proliferation. This study adds new evidence that highlights SVV is encapsulated in exosomes. Previous studies have shown that viruses carried by exosomes, such as PRRSV, HCV, and EV71, cannot be neutralized by neutralizing antibodies [16, 24, 32–35]. When SVV-exo was incubated with SVV neutralizing antibodies, the proliferation of SVV in exosomes was not affected by SVV neutralizing antibodies. This shows that SVV virus particles are encapsulated in exosomes.

In conclusion, we successfully extracted, purified, and identified exosomes from SVV-infected IBRS-2 cells and determined that exosomes can carry SVV, which allows the proliferation of the virus in susceptible and non-susceptible cells. The inhibition of exosome secretion and production inhibits the replication of SVV. Moreover, exosomes extracted from IBRS-2 cells promote the proliferation of SVV. SVV carried in exosomes was not blocked by SVV neutralizing antibodies. Collectively these data reveal an advanced and novel mechanism for better understanding that viral transmission through exosomes contributes to the known immune evasive properties of SVV.
