# Correction to: Intercellular transmission of Seneca Valley virus mediated by exosomes

**DOI:** 10.1186/s13567-020-00827-4

**Published:** 2020-08-18

**Authors:** Guowei Xu, Shouxing Xu, Xijuan Shi, Chaochao Shen, Junhong Hao, Minhao Yan, Dajun Zhang, Zixiang Zhu, Keshan Zhang, Haixue Zheng, Xiangtao Liu

**Affiliations:** State Key Laboratory of Veterinary Etiological Biology, National Foot-and-Mouth Disease Reference Laboratory, Lanzhou Veterinary Research Institute, Chinese Academy of Agriculture Science, Lanzhou, 73004 China

## Correction to: Vet Res (2020) 51:91 10.1186/s13567-020-00812-x

Following publication of the original article [1], the authors identified that the author Haixue Zheng should be corresponding author.
